# Variations in branching of the posterior cord of brachial plexus in a Kenyan population

**DOI:** 10.1186/1749-7221-6-1

**Published:** 2011-06-07

**Authors:** Johnstone M Muthoka, Simeon R Sinkeet, Swaleh H Shahbal, Ludia C Matakwa, Julius A Ogeng'o

**Affiliations:** 1 Department of Human Anatomy, University of Nairobi, P.O Box 30197,00100, Nairobi, Kenya

## Abstract

**Background:**

Variations in the branching of posterior cord are important during surgical approaches to the axilla and upper arm, administration of anesthetic blocks, interpreting effects of nervous compressions and in repair of plexus injuries. The patterns of branching show population differences. Data from the African population is scarce.

**Objective:**

To describe the branching pattern of the posterior cord in a Kenyan population.

**Materials and methods:**

Seventy-five brachial plexuses from 68 formalin fixed cadavers were explored by gross dissection. Origin and order of branching of the posterior cord was recorded. Representative photographs were then taken using a digital camera (Sony Cybershot ^R^, W200, 7.2 Megapixels).

**Results:**

Only 8 out of 75 (10.7%) posterior cords showed the classical branching pattern. Forty three (57.3%) lower subscapular, 8(10.3%) thoracodorsal and 8(10.3%) upper subscapular nerves came from the axillary nerve instead of directly from posterior cord. A new finding was that in 4(5.3%) and in 3(4%) the medial cutaneous nerves of the arm and forearm respectively originated from the posterior cord in contrast to their usual origin from the medial cord.

**Conclusions:**

Majority of posterior cords in studied population display a wide range of variations. Anesthesiologists administering local anesthetic blocks, clinicians interpreting effects of nerve injuries of the upper limb and surgeons operating in the axilla should be aware of these patterns to avoid inadvertent injury. A wider study of the branching pattern of infraclavicular brachial plexus is recommended.

## Background

The posterior cord of the brachial plexus usually gives upper subscapular, thoracodorsal, lower subscapular and axillary nerves in the axilla, continuing distally as the radial nerve [1]. Variations from this classical branching pattern differ in prevalence between populations [2-4]. In clinical practice, injuries to branches of the posterior cord are common and associated with each other [5]. Knowledge of possible variations may help in the management of such injuries. Further, understanding of the variations is valuable in the administration of anaesthetic blocks [4,6], surgical approaches to the neck, axilla and upper arm, interpretation of nervous compressions by tumours or aneurysms [4] and use of the subscapular branches in neurotization procedures for repair of plexus injuries due to birth trauma [7]. Literature on the variations of the posterior cord among African is scanty and altogether lacking in Kenyans. The present study describes the variations of the posterior cord observed in a black Kenyan population.

## Materials and methods

Brachial plexuses from sixty eight (33 male & 35 female) formalin fixed cadavers obtained from the Department of Human Anatomy, University of Nairobi were studied. Ethical approval was granted by the Kenyatta National Hospital/University of Nairobi Ethics and Research Committee. The age range of cadavers examined was 20-76 years. The upper limb was abducted and rotated laterally. Skin and superficial fascia in the chest wall were removed and pectoralis major and deltoid muscles detached from their origins. Clavipectoral fascia was cut near its clavicular attachment then pectoralis minor was detached from its origin and reflected upwards to expose the contents of the axilla. Axillary sheath was incised and connective tissue, fat and lymph nodes dissected away. Posterior cord was identified by its posterior relation to the axillary artery and by the branches arising from it. Origins and courses of all its branches were defined and recorded. Representative photographs were taken using a Sony Cybershot ^R ^(DSC W50, 7.2 MP) digital camera.

## Results

### Origin of branches

**Figure 1:**
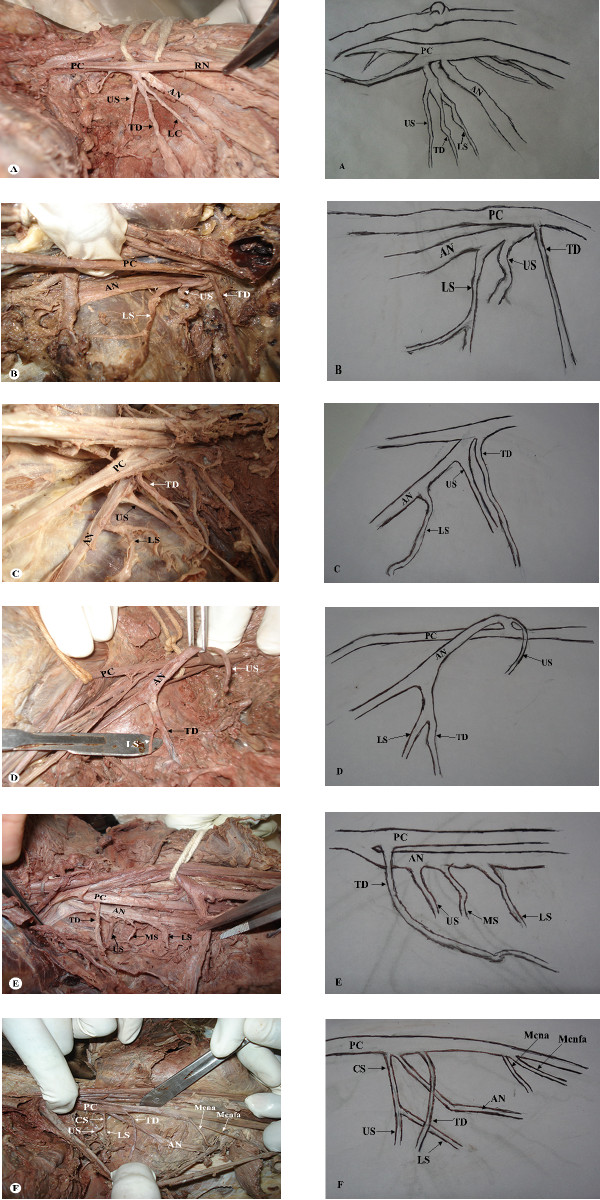
**Photomacrographs of axillary region showing posterior cord of the brachial plexus and its branches**. A: Common trunk giving rise to upper subscapular (US), thoracodorsal (TD) and lower subscapular (LS) nerves from the posterior cord (PC). Axillary nerve (AN) emerges as a separate branch. B: Supraclavicular origin of axillary nerve (AN) from posterior cord (PC). It gives off upper subscapular (US) and lower subscapular (LS) nerves. Thoracodorsal (TD) nerve emerges as a separate branch form the PC. C: Infraclavicular origin of axillary nerve (AN) from posterior cord (PC). It gives off upper subscapular (US) and lower subscapular (LS) nerves. Thoracodorsal (TD) nerve emerges as a separate branch form the PC. D: Axillary nerve (AN) from posterior cord (PC) giving a common trunk that separates into lower subscapular (LS) and thoracodorsal (TD) nerves. Upper subscapular (US) emerges separately from the AN. E: Axillary nerve (AN) giving upper subscapular (US), middle subscapular (MS) and lower subscapular (LS) nerves. Thoracodorsal (TD) nerve emerges separately from the posterior cord (PC). F: Common subscapular (CS) from the posterior cord (PC) that divides into upper subscapular (US) and lower subscapular (LS) nerves. Axillary nerve (AN) and Thoracodorsal (TD) emerge as separate branches from the posterior cord (PC). In this specimen, the PC further gave rise to the medial cutaneous nerve of the arm (Mcna) and medial cutaneous nerve of the forearm (Mcnfa).

The posterior cord of brachial plexus was formed from posterior divisions of brachial plexus in all specimens studied. In all except one case, the divisions joined above clavicle (Figure 1A). Radial nerves in all cases studied originated from the posterior cord as its terminal branch (Figure 1 A-F). Seventy three axillary nerves (97.3%) originated from the infraclavicular posterior cord while the remaining had a supraclavicular origin (Figure 1B). Thoracodorsal nerve originated from posterior cord in 66 (88%), axillary nerve in 8 (10.7%) and from a common trunk with upper and lower subscapular nerves in 1 (1.3%) specimen (Figure 1A). In one case, thoracodorsal nerve originated from a common trunk with lower subscapular nerve from among those from axillary nerve (Figure 1D). Upper subscapular nerve, on the other hand, was given off by posterior cord in 54 (72%), axillary nerve in 10%, (Figure 1C) and from a common trunk with lower subscapular nerve in one case. Twelve (16%) lower subscapular nerves branched from posterior cord, 43 (57.3%) from axillary nerve (Figure 1B-E), 9 (12%) from thoracodorsal nerve whereas it was absent in 9 brachial plexuses. A common subscapular trunk that gave upper and lower subscapular nerves was observed in 10 (13.3%) brachial plexuses (8 from posterior cord and 2 from axillary nerve). One axillary nerve in addition to being the source of upper and lower subscapular nerve, also gave an accessory/middle subscapular nerve (Figure 1E). Posterior cord also gave off the medial cutaneous nerve of the arm and forearm in 3 (4%) and 4 (5.3%) specimens respectively (Figure 1F).

### Order of branching

Eight of the 75(10.7%) posterior cords had the classical branching pattern i.e. upper sub-scapular(U), thoracodorsal (T), lower sub-scapular (L), axillary (A) and radial (R) nerves in that order. The commonest branching pattern was UTA(L)R* being seen in 32 of the 75 (42.7%) posterior cord branches followed by UTLAR (10.7%) and TA(UL)R (6.7%). Others had one variant from the classical pattern either in order or in number of individual branches. In 20 (26.7%) of cases, there were isolated variations such as TA(UL)R, T_1_A(UL)T_2_R, 2UA(LT)R, ULTAR, UT_1_A(LT_2_)R, 2UA(L)TR, UA(L)TR, 2ULA(T)R, T_1_UT_2_A(L)R, U_1_T(U_2_)LAR, UA(TL)R, Mcna** and MCnfa*** [Table 1]

**Table 1:** Order of branching of posterior cord

Order of branching	No	(%)
**UTA(L)R**	**32**	**(42.7)**

**UTLAR**	**8**	**(10.7)**

**TA(UL)R**	**5**	**(6.7)**

**TC(UL)AR**	**3**	**(4.0)**

**UT(L)AR**	**3**	**(4.0)**

**UTA(L)McnaR**	**2**	**(2.7)**

**TUA(L)R**	**2**	**(2.7)**

**Others**	**20**	**(26.7)**

**Total**	**75**	**(100)**

Note: The nerve within the brackets originated from the nerve preceding them. For example, in UTA(L)R, lower subscapular nerve originated from Axillary nerve.

Others: TA(UL)R, T_1_A(UL)T_2_R, 2UA(LT)R, ULTAR, UT_1_A(LT_2_)R, 2UA(L)TR, UA(L)TR, 2ULA(T)R, T_1_UT_2_A(L)R, U_1_T(U_2_)LAR, UA(TL)R, Mcna and MCnfa

A number preceding a letter means there were two branches of the nerve represented by the letter.

Abbreviations

A- Axillary nerve.

L- Lower subscapular nerve.

Mcna- Medial cutaneous nerve of the arm.

Mcnfa- Medial cutaneous nerve of the forearm.

R- Radial nerve.

T- Thoracodorsal nerve.

U- Upper subscapular.

**NB: **These variations were unilateral in nature.

* The nerve in brackets originated from the preceding nerve. For example, in UTA(L)R, lower subscapular nerve originated from Axillary nerve.

** Mcna- Medial cutaneous nerve of the arm.

*** Mcnfa- Medial cutaneous nerve of the forearm.

## Discussion

Classical order of branching was found in only 8 (10.7%) of the posterior cords while UTA (L)R was the most frequent order of branching seen in 32 (42.7%). The high incidence of variations in plexus patterns observed in this study may be due to unusual formation during the development of trunks, divisions, or cords [8]. Descriptions of peripheral nerve variations are useful in clinical and surgical practice, since an anatomical variation can be the cause of nerve palsy syndromes and vascular problems. They are of particular importance during diagnosis of injuries of the plexus, neck dissections, infraclavicular block procedures and surgical approaches to axillary region tumors where these unusual distributions are prone to damage. Further, identification of specific nerves originating from posterior cord of brachial plexus is necessary during neurotization processes [4,9,10].

In the current study, similar to conventional descriptions, radial nerves consistently originated from the posterior cord as its terminal branch [1,2]. This implies that it is a reliable landmark after which the other nerves can be identified. Axillary nerves originated from the posterior cord in 97.3% cases while two (2.7%) cases had a supraclavicular origin. This is important in nerve entrapment syndromes involving subclavius muscle and such supraclavicular axillary nerves. Axillary nerve has also been used as a landmark for identifying the lower subscapular nerve during glenohumeral joint surgery [11]. Accordingly, such variant positions could impact on the accuracy of such identification.

The thoracodorsal nerve was given from the posterior cord in 88% of cases. This is within the range of 78.6% and 98.5% described in literature [4,7]. The rest originated from the axillary nerve (10.7%) which was similar to 8.9% found by Ballesteros & Ramirez [12] but slightly lower than 13% reported by Fazan et al [3]. A hitherto unreported finding is that one thoracodorsal nerve originated from a common trunk with upper and lower subscapular nerves. Clinically, trauma of the posterior wall of the axillary region could present with a wide range of degrees of muscle impairment. Variations described here may explain these presentations which depend on lesion level and the degree of involvement of the thoracodorsal nerve's several origins. For instance, lesions involving axillary nerves that give rise to thoracodorsal nerve may produce more extensive functional lesions including latisimus dorsi, deltoid and teres minor muscles [5].

Subscapular nerves exhibited wide variations in origin and order of branching similar to literature reports [4]. Upper subscapular nerve originated from axillary nerve in 10 (13.3%), significantly higher than values reported by other studies [Table 2]. The nerve originated as a single nerve in 56 (74.6%) cases, 2 separate branches in 5 (6.7%) and as three trunks in one (1.3%) case. Lower subscapular nerve on the other hand, originated from the thoracodorsal nerve in 9 (12%) brachial plexuses which is similar to previous findings [Table 2]. Forty three (57.3%) lower subscapular nerves were given off by axillary nerve which is within range of 54-57.3% reported [3, 4, 13, 14, Table 2]. In 9(12%) specimens, the nerve originated from a common trunk with upper subscapular nerve. A new remarkable finding is that one brachial plexus lacked the upper subscapular nerve. This wide range of variation suggests population differences in anatomy of the brachial plexus. This variant anatomy is important in explaining the outcome of attempted subscapular block in hemiplegic patients with painful shoulder [15].

**Table 2:** Population variance of the incidence of axillary origin of the subscapular nerves

AUTHOR	POPULATION	Lower-subscapular from Axillary	Upper-subscapular from Axillary
**Ballesteros&Ramirez, 2000**	Colombian	54.4%	3.0%

**Tubbs et al., 2007**	American	21.0%	3.0%

**Kerr et al., 1918**	American	54.0%	-

**Fazan et al., 2003**	Brazil	54.0%	5.5%

**Current study **	Kenyan	57.3%	13.3%

In two cases the subscapular artery divided the posterior cord into two which then joined to form the radial nerve. This is concordant with a report by Kumar [6] in one case from 47 cadavers. This rare variation may be caused by the segmental origin of the axillary artery and its branching which may determine the arrangement of the brachial plexus during fetal development [16].

Knowledge of these variations is important to vascular surgeons working on this region. The posterior cord unusually gave origin to the medial cutaneous nerves of the forearm and arm in 3 (4%) and 4 (5.3%) plexuses respectively. these previously unreported findings are important in explaining outcome of anesthetic blocks and in interpreting nerve injuries.

## Conclusion

Majority of posterior cords in studied population display a wide range of variations. Anesthesiologists administering local anesthetic blocks, clinicians interpreting effects of nerve injuries to the upper limb and surgeons operating in the axilla should be aware of these patterns to ensure correct management and avoid inadvertent injury. A wider study of the branching pattern of infraclavicular brachial plexus is recommended.

## Competing interests

The authors declare that they have no competing interests.

## Authors' contributions

MM was involved in the conception and design of the study, data collection and analysis, drafting, revision and correction of the manuscript. SR was involved in data analysis, drafting, revision and correction of the manuscript. SS was involved in conception and design of the study, data collection and analysis, drafting of the manuscript. ML was involved in conception and design of the study, data collection and analysis, drafting of the manuscript. OJ was involved in revision and final approval of the manuscript version to be published. All authors have read and approved the final manuscript.
